# Dual inhibition of Akt and c‐Met as a second‐line therapy following acquired resistance to sorafenib in hepatocellular carcinoma cells

**DOI:** 10.1002/1878-0261.12039

**Published:** 2017-02-17

**Authors:** Peng Han, Hali Li, Xian Jiang, Bo Zhai, Gang Tan, Dali Zhao, Haiquan Qiao, Bing Liu, Hongchi Jiang, Xueying Sun

**Affiliations:** ^1^ The Hepatosplenic Surgery Center Department of General Surgery the First Affiliated Hospital of Harbin Medical University China; ^2^ Department of General Surgery the Fourth Affiliated Hospital of Harbin Medical University China

**Keywords:** acquired resistance, Akt, cellular signaling pathway, c‐Met, hepatocellular carcinoma, sorafenib

## Abstract

Sorafenib displays a limited efficacy for advanced hepatocellular carcinoma (HCC). Some patients with HCC initially respond to sorafenib, but eventually succumb to the disease, indicating that the acquired resistance to sorafenib reduces its beneficial effects. No alternative drugs are available after the failure of sorafenib therapy. Therefore, investigation of the mechanisms underlying the acquired resistance and development of second‐line treatments for sorafenib‐resistant HCC are urgently required. In this study, sorafenib‐resistant HCC cells generated from sorafenib‐sensitive human HCC cells were shown to overproduce hepatocyte growth factor (HGF) and overexpress c‐Met kinase and its phosphorylated form, leading to the activation of Akt and ERK (extracellular signaling‐regulated kinase) pathways. Use of specific c‐Met inhibitors enhanced the effects of sorafenib by inhibiting the growth of sorafenib‐resistant HCC cells. Akt inhibitors, a class of second‐line therapeutic drugs under investigation for treating HCC in clinical trials, enhanced the effects of sorafenib, but also activated the c‐Met pathway in sorafenib‐resistant cells. Dual inhibition of Akt and c‐Met by their respective inhibitors, MK2206 and capmatinib, additively or synergistically suppressed sorafenib‐resistant HCC cells *in vitro* and sorafenib‐resistant HCC xenografts in mice. The anticancer activities of MK2206 mainly rely on its ability to induce cell apoptosis and autophagic death, while capmatinib treatment leads to cell cycle arrest at phase G1. These results provide strong evidence for further investigation on the clinical utility of dual inhibition of Akt and c‐Met, particularly MK2206 and capmatinib, as a second‐line therapy for advanced HCC that has acquired resistance to sorafenib.

**Abbreviations**
4EBP1: initiation factor 4E‐binding protein 1
ERK: extracellular signaling‐regulated kinase
GSK3β: glycogen synthase kinase‐3β
HCC: hepatocellular carcinoma
HGF: hepatocyte growth factor
mTOR: mammalian target of rapamycin
PDGFR: platelet‐derived growth factor receptor
VEGFR: vascular endothelial growth factor receptor

## 1 Introduction

Hepatocellular carcinoma (HCC) is the third leading cause of cancer‐related death worldwide (Bruix *et al*., 2016). Sorafenib remains the unique systematic drug approved for the treatment of advanced HCC, although it prolongs the overall survival for only 2~3 months compared to placebo (Cheng *et al*., 2009; Llovet *et al*., 2008). Some patients with HCC initially respond to sorafenib, but eventually succumb to the disease, indicating that the acquired resistance to sorafenib greatly limits its beneficial effects (Waidmann and Trojan, 2015). Therefore, investigating the mechanisms underlying the acquired resistance to sorafenib and seeking second‐line therapies are urgently required. Unfortunately, until now no alternative agents have been demonstrated to be effective in treating HCC after the failure of sorafenib (Chan *et al*., 2016).

The hepatocyte growth factor (HGF)/c‐Met signaling axis is a potential target for cancer therapy. HGF is the known ligand of c‐Met (also called Met, hepatocyte growth factor receptor [HGFR]) and a paracrine factor, secreted predominantly by mesenchymal cells (Moran‐Jones *et al*., 2015). Binding of HGF to c‐Met leads to its homodimerization and autophosphorylation and subsequent activation of the ERK (extracellular signaling‐regulated kinase) and Akt pathways (Peters and Adjei, 2012). Activation of c‐Met promotes the proliferation, survival, and invasion of cells from various types of cancer (Blumenschein *et al*., 2012) including HCC (Giordano and Columbano, 2014). Although the HGF/c‐Met pathway remains idle in livers under physiological conditions, it is activated in HCC tissues and contributes to tumor biological aggressiveness and poor prognosis (Bupathi *et al*., 2015; Giordano and Columbano, 2014).

In particular, c‐Met activation confers chemotherapeutic resistance (Gherardi *et al*., 2012), and its inhibition reverses cisplatin resistance in ovarian cancer cells (Li *et al*., 2016). The c‐Met pathway is activated by small‐molecule inhibitors targeting anaplastic lymphoma kinase (Kogita *et al*., 2015) and epidermal growth factor receptor (Dulak *et al*., 2011) in non‐small‐cell lung cancer cells. The expression of c‐Met is elevated in trastuzumab‐ and vinorelbine‐resistant breast cancer tissues (Ho‐Yen *et al*., 2015). Sorafenib treatment also increases c‐Met expression, and inhibition of c‐Met synergizes with sorafenib to suppress HCC cells (Jiang *et al*., 2015). Specific c‐Met inhibitors, such as tivantinib (Rota Caremoli and Labianca, 2014; Santoro *et al*., 2013) and foretinib (Yau *et al*., 2012), have been clinically trialed in the treatment for HCC.

The Akt pathway is critically implicated in HCC progression by regulating myriad downstream targets (Hu *et al*., 2016; Manning and Cantley, 2007). We and others have previously reported that Akt activation contributes to sorafenib resistance of HCC cells (Piguet *et al*., 2011; Zhai *et al*., 2014; Zhang *et al*., 2016). Chronic exposure of HCC cells to sorafenib activates Akt, resulting in upregulation and/or activation of its downstream factors including ribosomal protein S6 kinase (S6K) and eukaryotic translation initiation factor 4E‐binding protein 1 (4EBP1) (He *et al*., 2015; Zhai *et al*., 2014). A number of Akt inhibitors have been developed and evaluated as a second‐line therapy for HCC (Yap *et al*., 2011). However, none of them have proven superior to sorafenib in treating HCC (Llovet and Hernandez‐Gea, 2014). Treatment of Akt inhibitors leads to tumor stasis instead of regression in preclinical models, and most patients either have partial or minimal responses from Akt inhibition in clinical trials (Llovet and Hernandez‐Gea, 2014). More disappointingly, partial inhibition of Akt even promotes the survival of cells from nonautonomous cancer through non‐cell‐autonomous communication (Salony *et al*., 2016).

Although Akt is regarded as a downstream factor of the c‐Met pathway (Peters and Adjei, 2012), co‐activation of Akt and c‐Met triggers in the progression of HCC (Hu *et al*., 2016). Here, we hypothesize that they could also cooperatively contribute to the mechanisms of the acquired resistance to sorafenib and their dual inhibition may represent a potential therapeutic strategy for advanced HCC, particularly as a second‐line treatment for those that are initially responsive, but eventually become resistant to sorafenib.

## 2 Materials and methods

### 2.1 Cells, antibodies, and reagents

Human HCC HepG2 cells were obtained from the American Type Culture Collection, and Huh7, MHCC‐7721, and MHCC‐3M cells from Chinese Academy of Sciences Cell Bank (Shanghai, China). Cells were cultured at 37 °C in Dulbecco's modified Eagle medium (DMEM) (Gibco BRL, Grand Island, NY, USA) supplemented with 10% fetal bovine serum. For details of antibodies and reagents, please refer to Appendix S1.

### 2.2 Sorafenib‐resistant cells

Sorafenib‐resistant HepG2 and Huh7 cells have been established previously (Zhai *et al*., 2014) and stored in liquid nitrogen. After resuscitation, cells were incubated with sorafenib at a starting concentration of 5 μm. The concentration of sorafenib was slowly increased by 1 μm per week. Cell viability was monitored weekly. After 1–2 months, two sorafenib‐resistant cell lines, termed HepG2‐SR and Huh7‐SR, were reobtained and continuously maintained by culturing them in the presence of sorafenib.

### 2.3 RT² Profiler™ PCR Array

The Human liver cancer RT² Profiler™ PCR Array (SABiosciences, Frederick, MD, USA) was employed to analyze the expression profiles of 84 key genes involved in HCC. The analysis was completed by KangChen Bio‐tech (Shanghai, China). Each assay was conducted in triplicate.

### 2.4 Quantitative reverse‐transcription polymerase chain reaction

Methods have been described in details previously (He *et al*., 2015; Zhai *et al*., 2014). Briefly, total RNA was extracted from cells, and cDNA was synthesized. The reaction mixtures for quantitative reverse‐transcription polymerase chain reaction (qRT‐PCR) were prepared with the primers for c‐Met mRNA (forward: 5′‐CTAGACACATTTCAATTGGT‐3′ and reverse: 5′‐TGTTGCAGGGAAGGAGTGGT‐3′, corresponding to nt 2262–2625 of human c‐Met [GenBank NM_000245.3]); HGF mRNA (forward: 5′‐ACCATGTGGGTGACCAAACT‐3′, reverse: 5′‐TGTGTTCGTGTGGTATCATGG‐3′, corresponding to nt 225–708 of human HGF [GenBank NM_001010932.2]); and an internal control GAPDH mRNA (forward: 5′‐ CACCCATGGCAAATTCCATGGCA‐3′ and reverse: 5′‐TCTAGACGGCAGGTCAGGTCCACC‐3′). The PCR products were analyzed by MX3000P Real‐time PCR systems (Stratagen, La Jolla, CA, USA). Experiments were performed in triplicate, and data were calculated by ∆∆Ct methods.

### 2.5 Animal experiments

Six‐ to eight‐week male BALB/c‐nu/nu mice were obtained from SLAC laboratory Animal Co., Ltd. (Shanghai, China). This study had been approved by the Animal Ethics Committee of Harbin Medical University, in compliance with the Experimental Animal Regulations by the National Science and Technology Commission, China (Permit SYXK20020009). The protocol has been described previously (He *et al*., 2015; Ma *et al*., 2014; Zhai *et al*., 2014). To examine whether Huh7‐SR cells continued to be sorafenib‐resistant *in vivo*, Huh7 or Huh7‐SR cells (5 × 10^6^) were subcutaneously inoculated into mice. Two weeks later when tumors grew to ~100 mm^3^, mice were assigned to vehicle or sorafenib groups. For second‐line therapy experiments, Huh7‐SR cells (5 × 10^6^) were subcutaneously inoculated into mice, which received daily oral administration of 10 mg·kg^−1^ sorafenib to maintain the sorafenib‐resistant ability of Huh7‐SR cells. The appearance of subcutaneous tumors was monitored. Twenty‐five days after cell inoculation, the mice bearing tumors of similar volumes (~100 mm^3^) were assigned to four groups: control, capmatinib, MK2206, and the combination therapy. Capmatinib and MK2206 were orally administered daily at a dose of 30 mg·kg^−1^ and 20 mg·kg^−1^, respectively. Mice in the control group received oral administration of vehicle. Tumors were measured every 4 days and harvested 20 days after the commencement of treatments.

### 2.6 Cell proliferation analysis, assessment of cell cycle and apoptosis *in vitro,* autophagy assays, transfection of Akt‐siRNA, enzyme‐linked immunosorbent assay, immunoblotting analysis, immunohistochemistry, *in situ* Ki‐67 proliferation index, and *in situ* detection of apoptotic cells

Above methods have been described previously (He *et al*., 2015; Ma *et al*., 2014; Zhai *et al*., 2014). Please refer to Appendix S1.

### 2.7 Statistical analysis

The data were expressed as mean values ± standard deviation. Comparisons were made using one‐way ANOVA followed by Dunnett's *t*‐test. *P* < 0.05 was considered significant.

## 3 Results

### 3.1 The c‐Met pathway is activated in sorafenib‐resistant HCC cells

**Figure 1:**
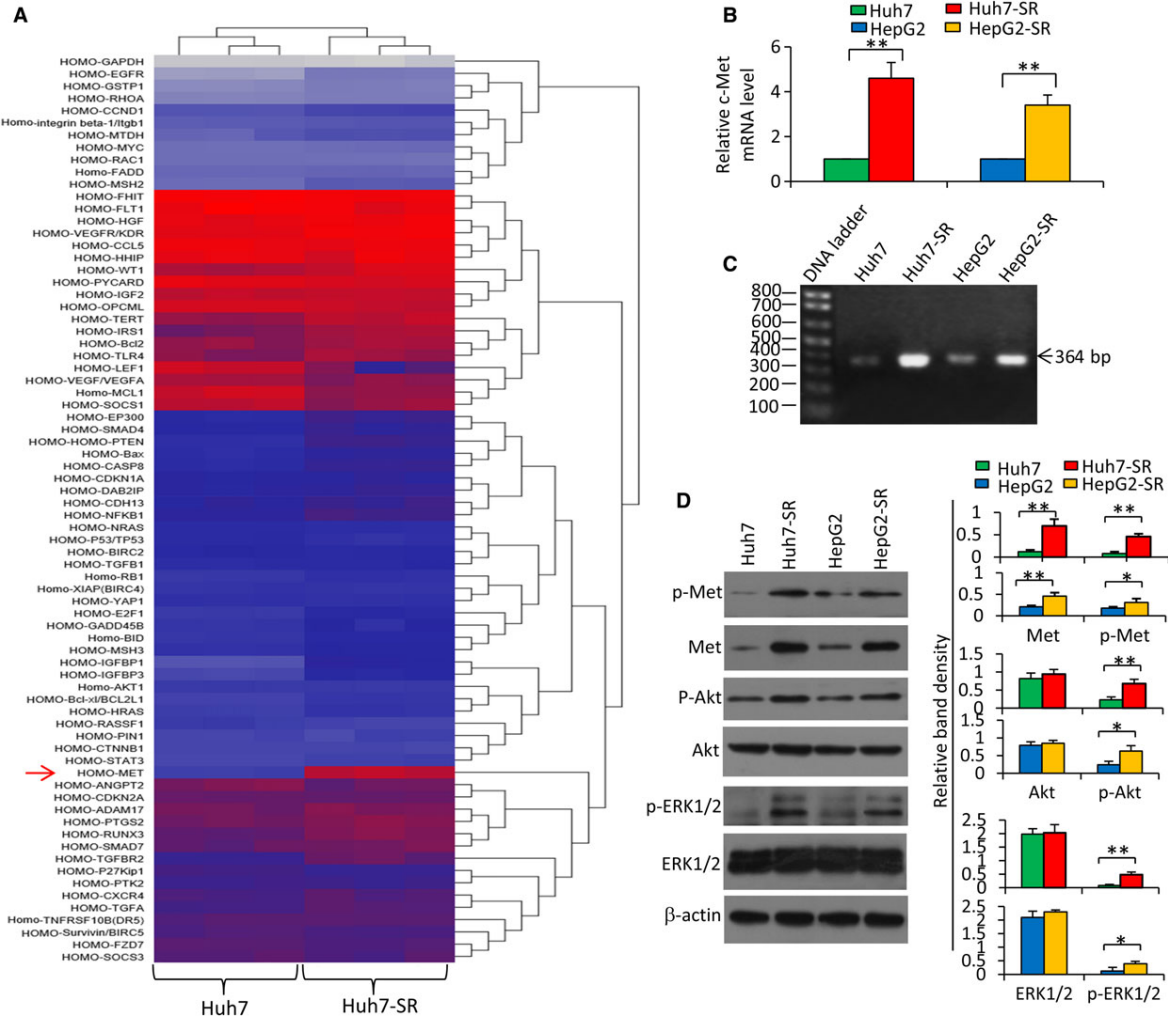
Sorafenib‐resistant HCC cells express higher levels of c‐Met. Huh7, Huh7‐SR, HepG2, and HepG2‐SR cells were cultured for 48 h and harvested for analysis. (A) The hierarchical clustering analysis of differentially expressed genes was performed by using a Human liver cancer RT² Profiler™ PCR Array on Huh7 and Huh7‐SR cells. (B) The expression of c‐Met mRNA was measured by qRT‐PCR. The level of mRNA from parental cells was defined as 1. (C) PCR products (364 bp) generated from RT‐PCR for detecting c‐Met mRNA underwent a 2% agarose gel electrophoresis. (D) The above cells were immunoblotted. The density of each band was normalized to β‐actin. ‘*’ (*P* < 0.05) and ‘**’ (*P* < 0.001) indicate a significant difference.

Sorafenib‐resistant HCC cells generated from sorafenib‐sensitive human HCC cells were shown to be refractory to sorafenib‐induced growth inhibition and apoptosis *in vitro* (Fig. S1), in agreement with our previous studies (He *et al*., 2015; Zhai *et al*., 2014). The alterations of gene expression were screened by a Human liver cancer RT² Profiler™ PCR Array, which showed that the expression of c‐Met mRNA was highly upregulated in Huh7‐SR cells compared with Huh7 cells (Fig. 1A). The raised levels of c‐Met mRNA were verified by qRT‐PCR (Fig. 1B) and conventional RT‐PCR (Fig. 1C) in Huh7‐SR and HepG2‐SR cells, compared with Huh7 and HepG2 cells, respectively. The expression of c‐Met and phosphorylated c‐Met (p‐Met) proteins showed a similar pattern to c‐Met mRNA (Fig. 1D). The expression levels of p‐Akt and p‐ERK, two major downstream factors of the c‐Met pathway (Peters and Adjei, 2012), were also elevated in sorafenib‐resistant cells (Fig. 1D).

### 3.2 Sorafenib activates the HGF/c‐Met and PTEN/Akt pathways in HCC cells

**Figure 2:**
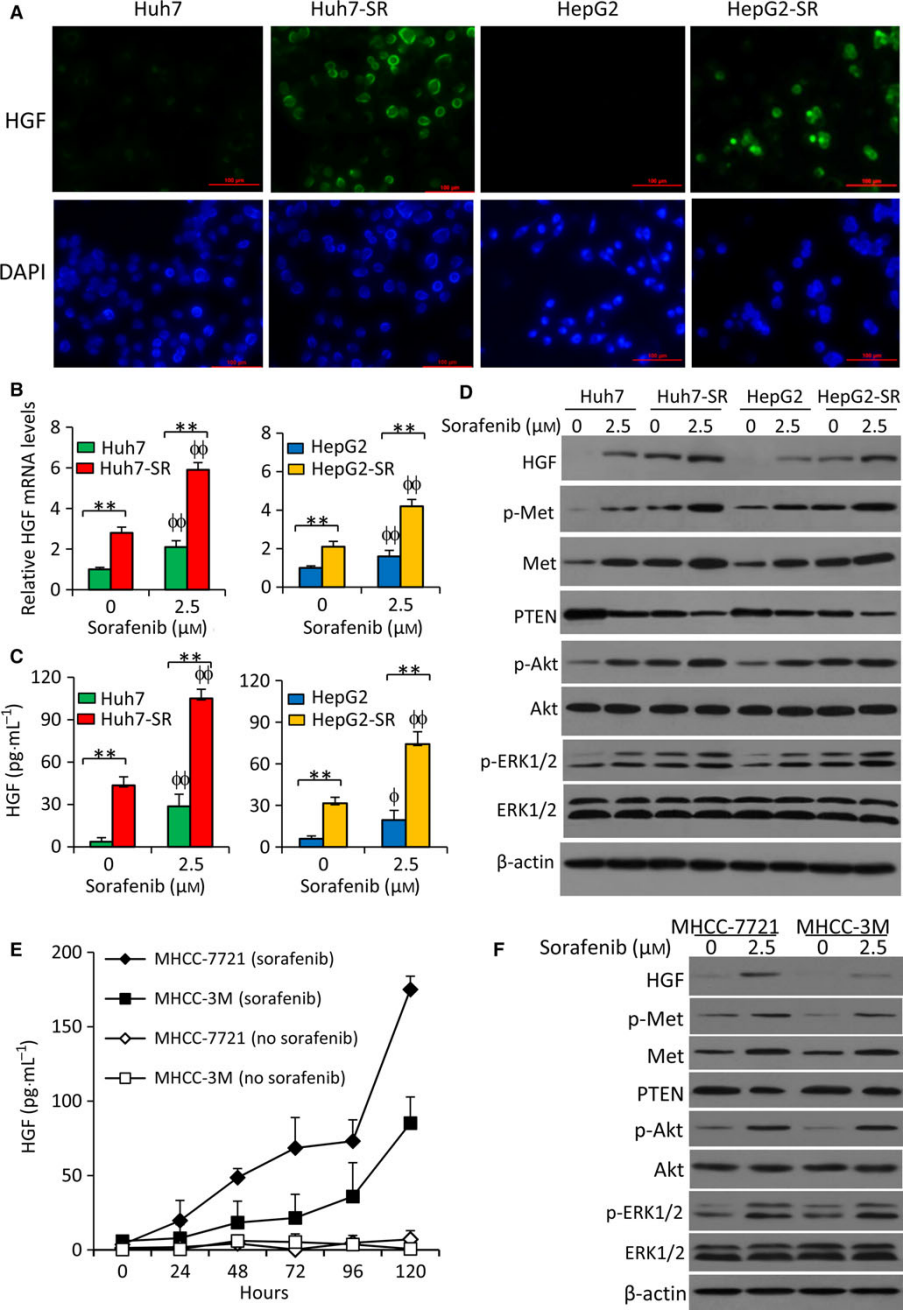
Exposure to sorafenib activates the HGF/c‐Met and PTEN/Akt pathways in HCC cells. (A) Cellular expression of HGF was detected by immunofluorescence microscopy. HGF protein was stained by an anti‐HGF antibody (green), and the cell nuclei were stained blue by DAPI. (B–D) Cells were incubated in the absence or presence of sorafenib (2.5 μm) for 48 h. (B) The expression of HGF mRNA was measured by qRT‐PCR. The level of mRNA from untreated parental cells was defined as 1. (C) The levels of HGF protein in the culture media were measured by ELISA. (D) The above cells were subjected to immunoblotting. (E, F) MHCC‐7721 and MHCC‐3M cells were incubated in the absence or presence of sorafenib (2.5 μm) and harvested at indicated time points. (E) The levels of HGF protein in the culture media were measured by ELISA. (F) Cells harvested at 48 h were immunoblotted. ‘**’ (*P* < 0.001) indicates a significant difference. ‘ϕ’ (*P* < 0.05) and ‘ϕϕ’ (*P* < 0.001) indicate a significant difference from respective untreated cells.

Sorafenib‐resistant HCC cells expressed higher levels of HGF than parental cells as detected by immunocytochemistry (Fig. 2A). Exposure of sorafenib (2.5 μm) for 48 h increased the expression of HGF mRNA (Fig. 2B) and the secretion of HGF protein (Fig. 2C) of both parental and sorafenib‐resistant cells. The elevated HGF production was confirmed by immunoblotting analysis (Fig. 2D). Sorafenib exposure resulted in upregulation of p‐Met, c‐Met, p‐Akt, and p‐ERK, and downregulation of PTEN, in both parental and sorafenib‐resistant HCC cells (Fig. 2D). Similar expression patterns of the above proteins were also observed in another two human HCC cell lines, MHCC‐7721 and MHCC‐3M (Fig. 2E,F).

### 3.3 Inhibition of c‐Met enhanced the effects of sorafenib to suppress sorafenib‐resistant HCC cells

**Figure 3:**
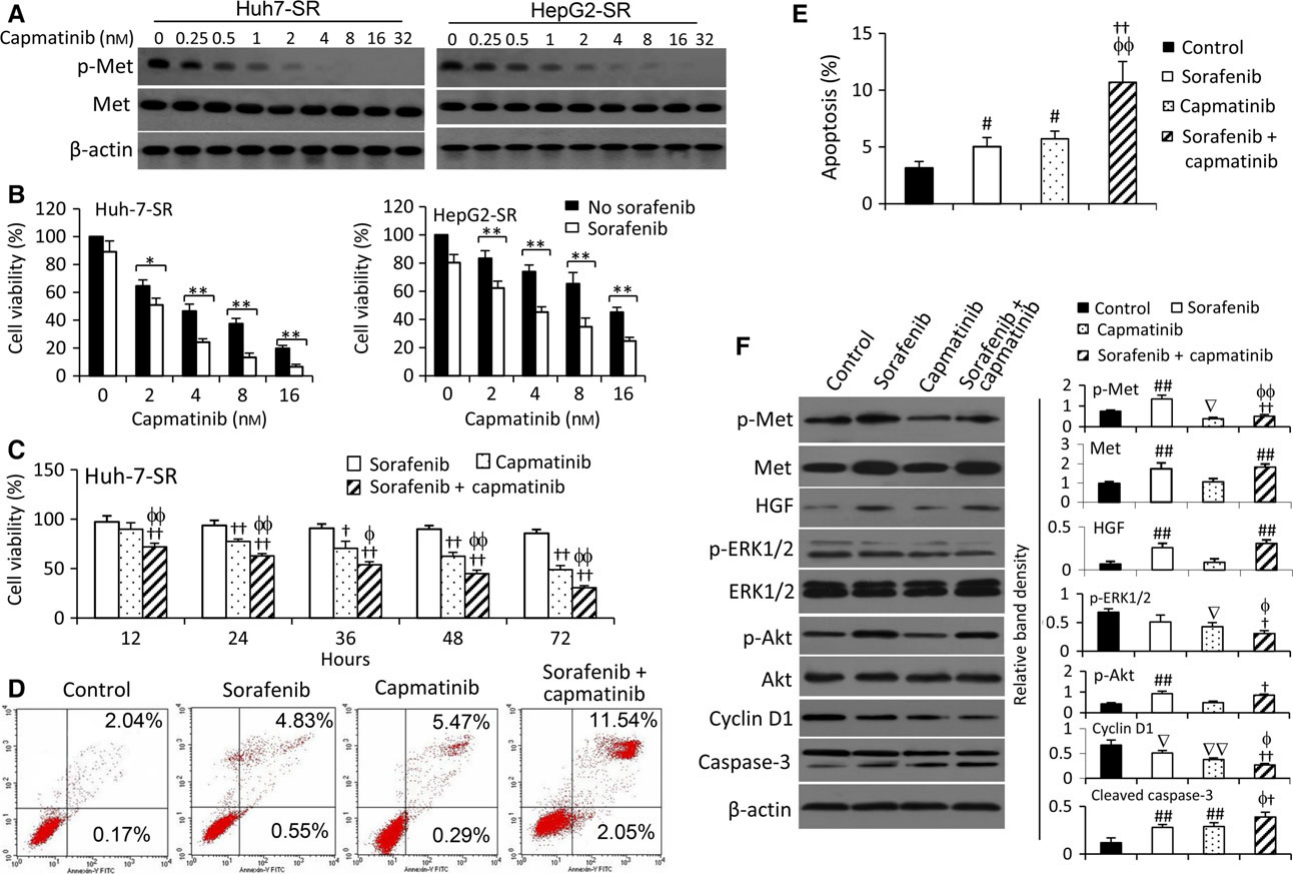
Inhibition of c‐Met by capmatinib enhances the sensitivity of sorafenib‐resistant HCC cells to sorafenib. (A, B) Huh7‐SR and HepG2‐SR cells were incubated for 48 h with various concentrations of capmatinib and subjected to immunoblotting (A) or cell viability assays (B). (C) Cells were incubated for 48 h with various concentrations of capmatinib in the presence or absence of sorafenib (5 μm). (D) Huh7‐SR cells were incubated with sorafenib (5 μm), capmatinib (2 nm), or the combination, and harvested at indicated time points. (B–D) Cell viability (%) was normalized to the respective untreated cells. (E–G) Huh7‐SR cells were incubated for 48 h with sorafenib (5 μm), capmatinib (2 nm), or the combination, and subjected to cytometry for measuring cell apoptosis (%) (E, F), or to immunoblotting analysis (G). The density of each band was normalized to β‐actin. ‘*’ (*P* < 0.05) and ‘**’ (*P* < 0.001) indicate a significant difference. ‘†’ (*P* < 0.05) and ‘††’ (*P* < 0.001) vs. sorafenib alone; ‘ϕ’ (*P* < 0.05) and ‘ϕϕ’ (*P* < 0.001) vs. capmatinib alone. ‘#’ (*P* < 0.05) and ‘##’ (*P* < 0.001) indicate a significant increase, while ‘∇’ (*P* < 0.05) and ‘∇∇’ (*P* < 0.001), a significant reduction, versus controls.

Incubation of capmatinib, a specific c‐Met inhibitor (Krepler *et al*., 2016), significantly downregulated the expression of p‐Met in a concentration‐dependent manner, but had no effect on c‐Met expression, in sorafenib‐resistant cells (Fig. 3A). Capmatinib significantly reduced the viability and strengthened the effects of sorafenib on sorafenib‐resistant cells in concentration‐ and time‐dependent manners (Fig. 3B,C), but only showed weaker effects on parental cells compared with respective sorafenib‐resistant cells (Fig. S2A). Capmatinib also enhanced the proapoptotic activity of sorafenib (Fig. 3D,E). The above results were supported by the experiments with cabozantinib, another c‐Met inhibitor (Fig. S3). Capmatinib was shown to correct the upregulated p‐Met and p‐Akt induced by sorafenib, and displayed an additive effect with sorafenib in downregulating p‐ERK1/2, cyclin D1, and activated caspase‐3 (Fig. 3F).

### 3.4 Inhibition of Akt activates the c‐Met pathway in sorafenib‐resistant HCC cells

**Figure 4:**
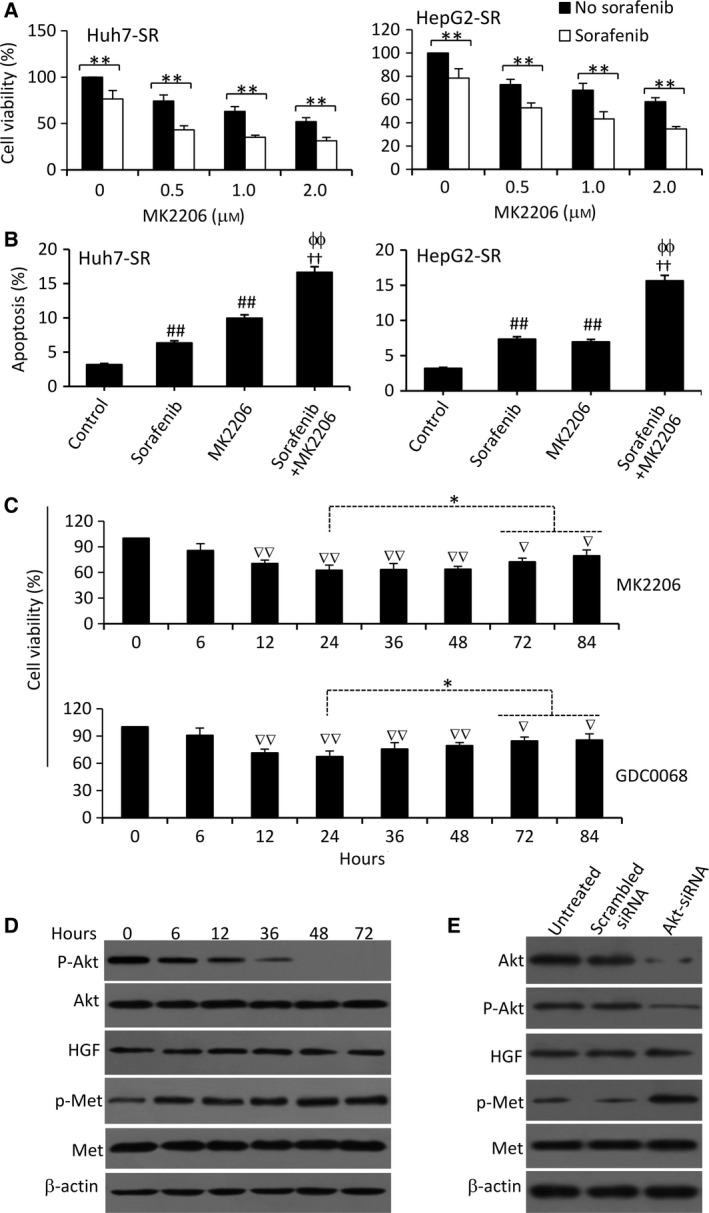
Inhibition of Akt suppresses sorafenib‐resistant HCC cells and activates the c‐Met pathway. (A) Huh7‐SR and HepG2‐SR cells were incubated for 48 h with various concentrations of MK2206 in the presence or absence of sorafenib (5 μm). Cell viability (%) was normalized to the respective untreated cells. (B) Cells were incubated for 48 h with sorafenib (5 μm), MK2206 (1 μm), or the combination. Untreated cells served as controls. Cell apoptosis (%) was measured by cytometry. (C) Huh7‐SR cells were incubated in media containing MK2206 (0.5 μm) or GDC0068 (5 μm), which were refreshed every 24 h. Cell viability (%) was measured at indicated time points and normalized to untreated cells. (D) Cells treated with MK2206 in (C) were immunoblotted. (E) Huh7‐SR cells were transfected with scrambled siRNA or Akt‐siRNA for 48 h and then subjected to immunoblotting. ‘*’ (*P* < 0.05) and ‘**’ (*P* < 0.001) indicate a significant difference. ‘††’ (*P* < 0.001) vs. sorafenib alone; ‘ϕϕ’ (*P* < 0.001) vs. MK2206 alone. ‘##’ (*P* < 0.001) indicates a significant increase, while ‘∇’ (*P* < 0.05) and ‘∇∇’ (*P* < 0.001), a significant reduction, versus controls.

GDC0068, an ATP‐competitive pan‐Akt inhibitor, has been shown to enhance the efficacy of sorafenib to suppress sorafenib‐resistant HCC cells (Zhai *et al*., 2014). MK2206, a novel selective inhibitor of pan‐Akt (Stottrup *et al*., 2016), was tested in the present study. In concentration‐dependent manners, MK2206 significantly increased the activity of sorafenib in reducing the viability (Fig. 4A) and inducing the apoptosis (Fig. 4B) of sorafenib‐resistant HCC cells, but only showed weaker effects on parental cells compared to respective sorafenib‐resistant cells (Fig. S2B). Interestingly, MK2206 did not show an inhibitory effect on the viability of Huh7‐SR cells in a time‐dependent manner. The inhibitory effect of MK2206 reached a peak at 24 h and prolonged incubation did not further reduce cell viability (Fig. 4C). The viability of cells incubated with 0.5 μm of MK2206 for 72 and 84 h was even slightly higher than that for the 24‐h incubation (Fig. 4C). A similar trend was found with GDC0068 (0.5 μm) (Fig. 4C). These results suggested that sorafenib‐resistant HCC cells became resistant to Akt inhibitors. In exploring the mechanisms, we found that MK2206 downregulated p‐Akt expression as expected, but also upregulated p‐Met expression in a time‐dependent manner (Fig. 4D). Supportively, depletion of Akt by siRNA also upregulated the expression of p‐Met, but had no effect on the expression of HGF and Met, in sorafenib‐resistant cells (Fig. 4E).

### 3.5 Dual inhibition of Akt and c‐Met suppresses sorafenib‐resistant HCC cells *in vitro*

**Figure 5:**
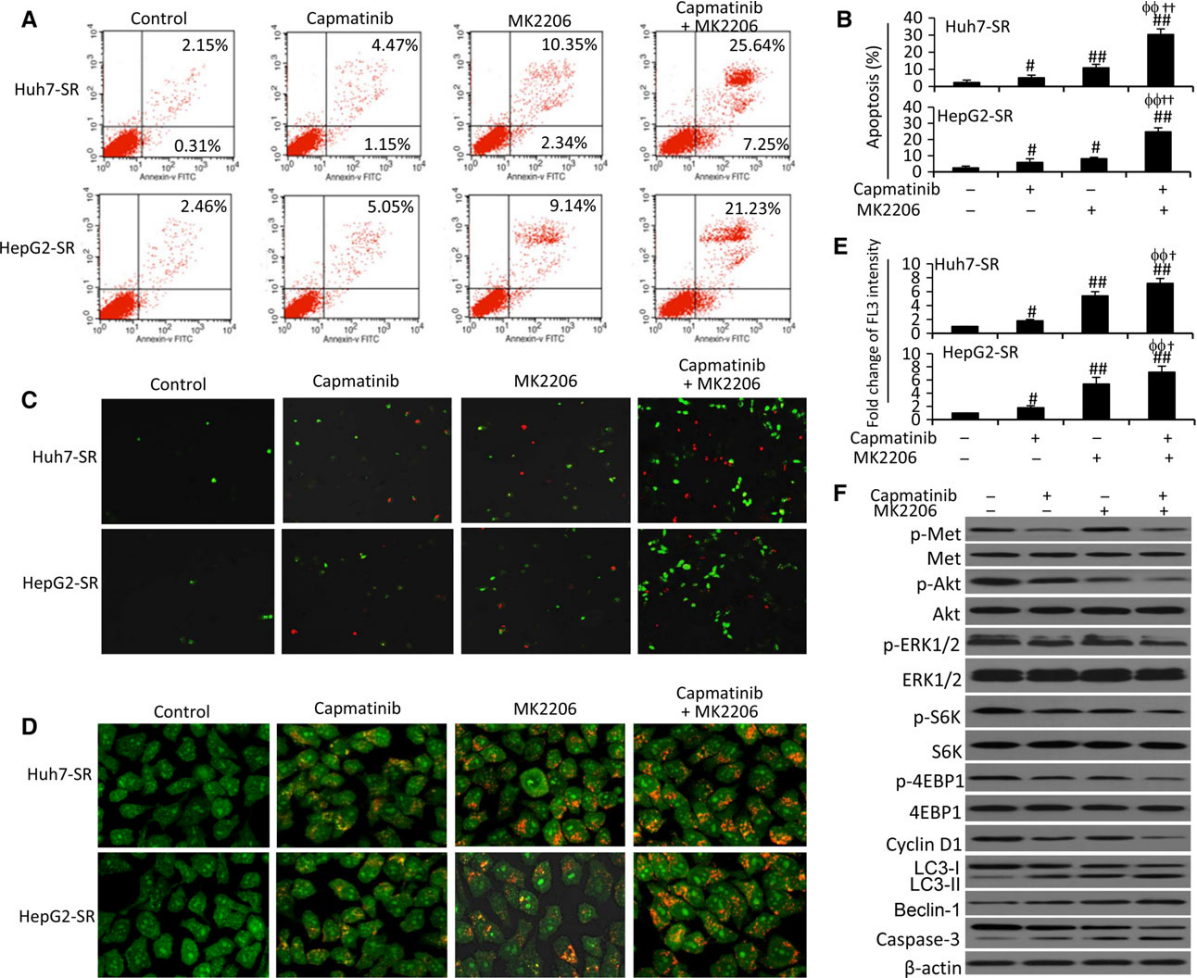
Dual inhibition of Akt and c‐Met induces apoptosis and autophagy of sorafenib‐resistant HCC cells. Huh7‐SR and HepG2‐SR cells were incubated for 48 h with capmatinib (2 nm), or MK2206 (1 μm), or the combination. (A, B) Cell apoptosis (%) was measured by cytometry. (C) Representative images were taken from cells stained with annexin V/propidium iodide (magnification ×100). (D) Representative images were taken from acridine orange‐stained cells (magnification ×400). (E) The fold change of acridine orange fluorescence intensity (FL3) versus untreated controls was calculated. The FL3 in untreated controls was defined as 1. (F) Lysates of the above Huh7‐SR cells were immunoblotted. ‘#’ (*P* < 0.05) and ‘##’ (*P* < 0.001) indicate a significant increase from controls. ‘ϕϕ’ (*P* < 0.001) vs. capmatinib alone; ‘†’ (*P* < 0.05) and ‘††’ (*P* < 0.001) vs. MK2206 alone.

The above results had driven us to investigate whether dual inhibition of c‐Met and Akt could suppress sorafenib‐resistant HCC cells. Both capmatinib and MK2206 induced apoptosis, but MK2206 exhibited a stronger proapoptotic activity than capmatinib, in sorafenib‐resistant cells (Fig. 5A–C). The values for the coefficient of drug interaction (CDI) (Wang *et al*., 2011; Zhai *et al*., 2014) for HepG2‐SR and Huh7‐SR cells were 0.67 and 0.61, respectively, indicating that two agents had a synergistic effect in inducing apoptosis (Fig. 5A–C). In addition, capmatinib, MK2206, or their combination showed weaker effects in inducing apoptosis of parental cells compared to respective sorafenib‐resistant cells (Fig. S2C). Autophagy plays a death‐promoting role in sorafenib‐resistant HCC cells (He *et al*., 2015; Zhai *et al*., 2014). Here, we showed that MK2206 treatment resulted in significantly more acridine orange‐stained acidic vesicular organelles (AVOs), while capmatinib induced a slight increase in AVOs (Fig. 5D). Quantitative analysis confirmed that MK2206‐treated cells had significantly higher fluorescence intensity (FL3), and the two agents showed an addictive effect as the values of CDI were 0.91 and 0.85 for Huh7‐SR and HepG2‐SR cells, respectively (Fig. 5E). The autophagic results were further supported by staining the above cells with monodansylcadaverine (MDC), a marker for autophagic vacuoles (Fig. S4). With regard to cell proliferation, capmatinib displayed a stronger inhibitory activity than MK2206 for both parental and sorafenib‐resistant cells, and they also showed weaker inhibitory effects on parental cells than on sorafenib‐resistant cells (Fig. S5A). In addition, capmatinib induced cell cycle arrest at phase G1 in sorafenib‐resistant cells (Fig. S5B,C).

Capmatinib treatment reduced the expression of p‐Met, p‐Akt, p‐ERK1/2, p‐S6K, p‐4EBP1, and cyclin D1, and increased caspase‐3 activation, but had little effect on the conversion of LC3‐1 to LC3‐II and Beclin‐1 expression (Fig. 5F). On the other hand, MK2206 reduced the expression of p‐Akt, p‐S6K, and p‐4EBP1, increased the conversion of LC3‐1 to LC3‐II, caspase‐3 activation, and p‐Met expression, but had a slight effect on the expression of p‐ERK1/2 and cyclin D1 (Fig. 5F). In cells treated with the combination of the two agents, capmatinib corrected MK2206‐induced p‐Met upregulation, and further reduced the expression of p‐Akt, p‐S6K, and p‐4EBP1 and increased caspase‐3 activation (Fig. 5F).

### 3.6 Dual inhibition of Akt and c‐Met suppresses sorafenib‐resistant tumors *in vivo*

**Figure 6:**
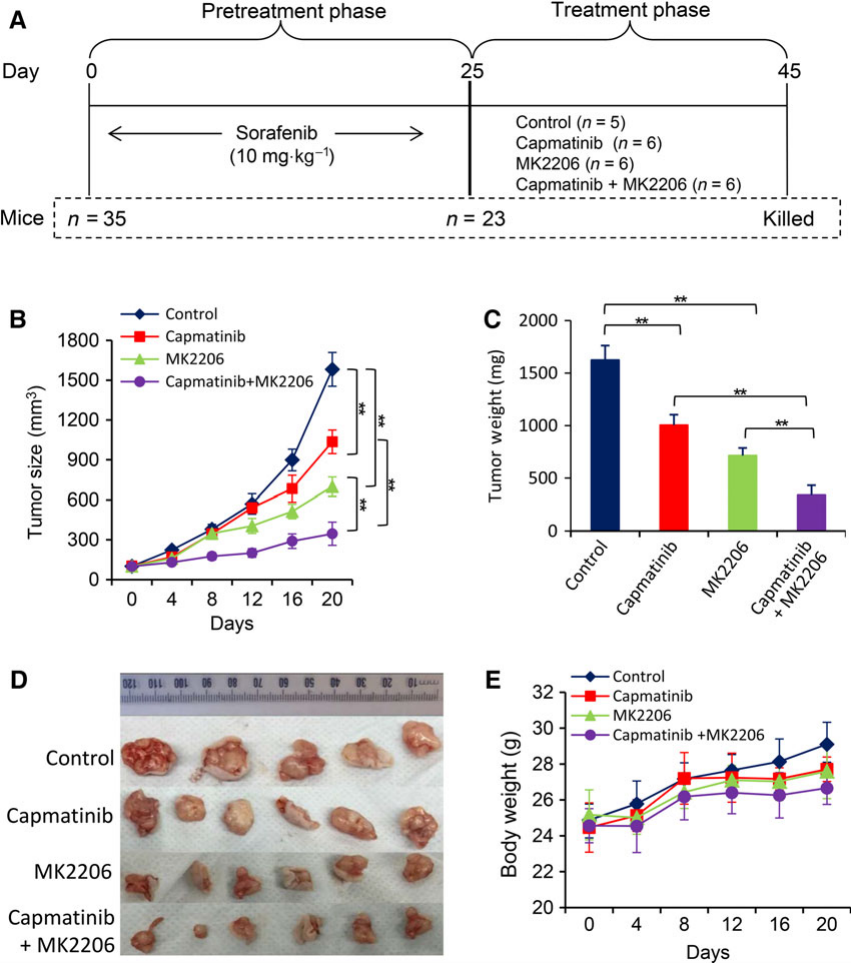
Dual inhibition of Akt and c‐Met as a second‐line therapy suppresses sorafenib‐resistant HCC tumors. (A) Animal experimental schedule was described in Materials and Methods. (B) The size (mm^3^) of tumors was recorded. Tumors harvested at the end of experiments were weighed (C) and photographed (D). (E) The bodyweights of mice were monitored ‘**’ (*P* < 0.001) indicates a significant difference.

Sorafenib‐resistant HCC cells were shown to be resistant to sorafenib *in vivo* (Fig. S6A), in agreement with our previous study (Zhai *et al*., 2014). For second‐line therapies, Huh7‐SR cells were inoculated into mice, which received daily administration of 10 mg·kg^−1^ sorafenib until tumors reached ~100 mm^3^, and were then assigned to different treatments (Fig. 6A). Huh7‐SR tumors continued to be insensitive to sorafenib treatment (Fig. S6B). However, administration of capmatinib and MK2206 significantly reduced the size of tumors by 34.5% and 55.8%, respectively, while the combination therapy resulted in a further reduction by 78.1%, compared with control tumors, 20 days after the commencement of treatments (Fig. 6B). The results were supported by the weights (Fig. 6C) and photographs (Fig. 6D) of tumors, which were harvested at the end of experiments. Either capmatinib or MK2206 or their combination showed little effects on bodyweight of mice (Fig. 6E).

The results were supported by *in situ* detection of cell proliferation by immunohistochemistry with an anti‐Ki67 antibody, and apoptosis by TUNEL staining (Fig. S7A,B). Capmatinib exhibited a stronger proliferation inhibitory ability than MK2206, while MK2206 had a more powerful proapoptotic activity than capmatinib. The two agents showed an additive effect in inhibiting cell proliferation, and a synergistic effect in promoting apoptosis *in situ*, as the values of CDI for proliferation and apoptosis indexes were 0.81 and 0.67, respectively. Immunohistochemical analyses of tumor tissues demonstrated similar alterations in the expression of p‐Met, p‐Akt, and cleaved caspase‐3 proteins (Fig. S7A), compared with that shown *in vitro* (Fig. 5F).

## 4 Discussion

Most patients with HCC have lost the opportunity for curative treatments at the time of diagnosis. Although several adjuvant therapeutic options are available, none of them are able to significantly improve the survival of patients with HCC after surgery according to a retrospective analysis from Cochrane databases (Samuel *et al*., 2009), indicating that drug therapy is irreplaceable in the treatment for HCC. Unfortunately, HCC is extremely resistant to conventional chemotherapy, and no standard chemotherapeutic regimens are available so far (Ang *et al*., 2013). The launch of sorafenib in 2007 opened a new era of molecular targeted therapy for HCC (Cheng *et al*., 2009; Llovet *et al*., 2008). Since then, 25 molecular targeted drugs have been investigated for HCC in phase III clinical trials, according to Medical Information Database (www.thomson-pharma.com). However, none of these drugs apart from sorafenib have reached the primary endpoints (Chan *et al*., 2016).

Increased expression of c‐Met is observed in over 80% of HCC tissues, and correlates with poor prognosis (Firtina Karagonlar *et al*., 2016; Jiang *et al*., 2015). Importantly, more evidence has indicated that the c‐Met signaling pathway might be a common mechanism of resistance to antiangiogenic drugs, such as sorafenib (Maroun and Rowlands, 2014). The activation of c‐Met is associated with chemoresistance in several types of cancer (Huang *et al*., 2016; Li *et al*., 2016) and participates in conferring the resistance to molecular targeted therapies (Firtina Karagonlar *et al*., 2016; Gou *et al*., 2016; Heynen *et al*., 2014; Kogita *et al*., 2015; Phan *et al*., 2015; Wilson *et al*., 2012). In the present study, we found that c‐Met was markedly upregulated in sorafenib‐resistant HCC cells, and sorafenib exposure increased the production of HGF and phosphorylation of c‐Met, leading to the activation of Akt and ERK pathways. Phosphatidylinositol 3‐kinase (PI3K), a downstream molecule of c‐Met and an upstream factor for Akt, is shown to be involved in sorafenib resistance of HCC (Ohta *et al*., 2015; Serova *et al*., 2013; Zhang *et al*., 2016), indicating that c‐Met may also regulate Akt activation through PI3K, but it needs further investigation. In support of our results, sorafenib was shown to upregulate c‐Met (Jiang *et al*., 2015), and long‐term sorafenib exposure increased the synthesis and secretin of HGF (Firtina Karagonlar *et al*., 2016). Inhibition of c‐Met by capmatinib (Krepler *et al*., 2016) and cabozantinib (Xiang *et al*., 2014) resulted in proliferation inhibition and apoptosis by downregulating cyclin D1 and activating caspase‐3. DE605, a c‐Met inhibitor, acts synergistically with sorafenib to suppress HCC cells (Jiang *et al*., 2015); SU11274, another c‐Met inhibitor, and HGF‐neutralizing antibody reversed the invasion properties of sorafenib‐resistant HCC cells (Firtina Karagonlar *et al*., 2016).

The Akt pathway plays a critical role in the mechanisms of sorafenib resistance by cross‐talking with the major sorafenib‐targeted ERK pathway (Aksamitiene *et al*., 2012). Although a number of Akt inhibitors have been developed and clinically evaluated (Yap *et al*., 2011), their clinical benefits are likely very limited due to the development of acquired resistance (Llovet and Hernandez‐Gea, 2014). Activation of receptor tyrosine kinases is implicated in acquisition of resistance to Akt inhibitors by relieving mTOR‐mediated feedback (Chandarlapaty *et al*., 2011). Compensatory feedback activation of ERK has been observed in tumor biopsies from patients treated with GDC0068 (Yan *et al*., 2013). Inhibition of Akt by either GDC0068 (Zhai *et al*., 2014) or MK2206 was able to transiently inhibit sorafenib‐resistant HCC cells, but also upregulated the expression of p‐Met. Therefore, co‐application of c‐Met inhibitors could enhance the effects of Akt inhibition in suppressing sorafenib‐resistant HCC cells. This proposal is supported by previous findings that co‐activation of Akt and c‐Met triggers the rapid development of HCC (Hu *et al*., 2016).

Unfortunately, the overexpression of Akt and c‐Met has not been confirmed in clinical sorafenib‐resistant HCC specimens, and the therapeutic effects of dual inhibition of Akt and Met have not been evaluated in patient‐derived xenografts, which are the limitations of the present study. As indicated in the guideline of American Association for the Study of Liver Diseases (AASLD), sorafenib is administered to patients with advanced HCC (Bruix *et al*., 2016). Those patients with late‐stage HCC have lost the chance for curative treatments, and cannot benefit from either laparotomy or needle biopsy, making it very difficult to collect clinical sorafenib‐resistant HCC tissues. We have not been fortune to collect these precious tissues by postmortem, which may be a possible way.

**Figure 7:**
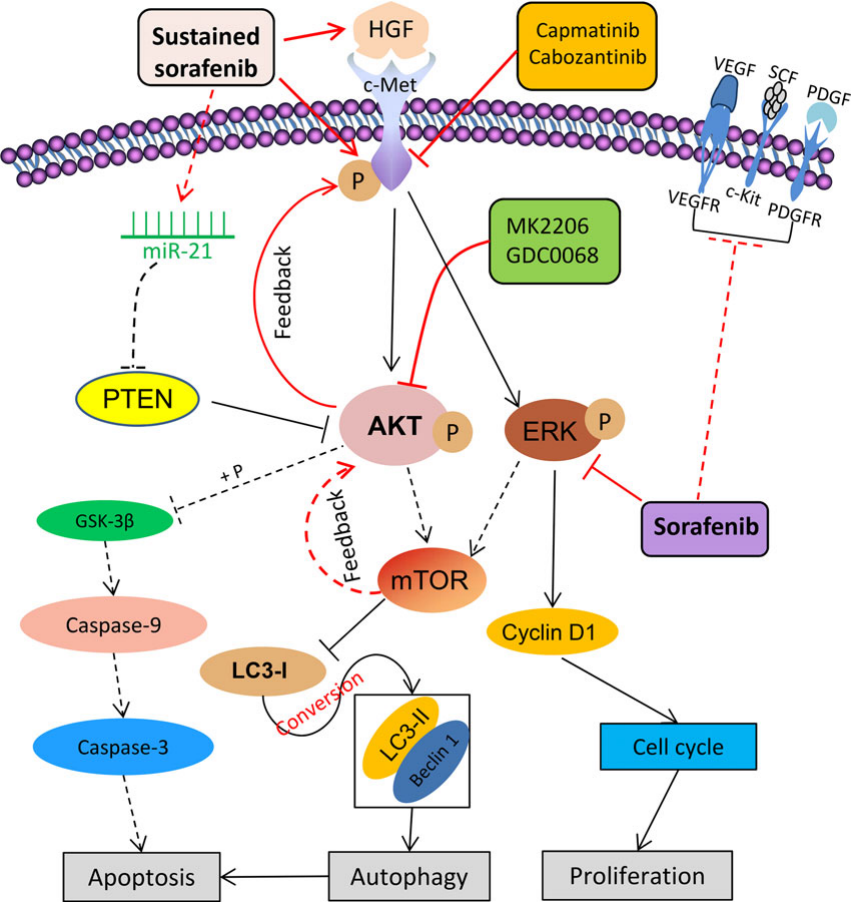
Proposed molecular mechanisms by which dual inhibition suppresses sorafenib‐resistant HCC cells by targeting the Akt and c‐Met pathways. ‘→’ indicates positive regulation or activation; ‘⊥’, negative regulation or blockade. Solid lines ‘____’ indicate mechanisms examined in the present study, while dotted lines ‘‐‐‐‐‐‐’, mechanisms in previous studies (He *et al*., 2015; Llovet *et al*., 2008; Zhai *et al*., 2014). Abbreviations and explanations: c‐Kit, also called stem cell growth factor receptor or CD117; c‐Met, also called hepatocyte growth factor receptor [HGFR]; ERK, extracellular signaling‐regulated kinase; GSK‐3β, glycogen synthase kinase‐3β; HGF, hepatocyte growth factor; LC3, microtubule‐associated protein 1 light chain 3; mTOR, mammalian target of rapamycin; PDGF, platelet‐derived growth factor; PDGFR, platelet‐derived growth factor receptor; PTEN, phosphatase and tensin homolog; SCF, stem cell factor; VEGF, vascular endothelial growth factor; VEGFR, vascular endothelial growth factor receptor.

The mechanisms for the acquired resistance to sorafenib remain highly complex (Berasain, 2013). The signaling pathways and their interactions in sorafenib‐resistant HCC cells, and the interventions with agents used in the present study, are depicted in Fig. 7. Sorafenib targets the ERK pathway and a number of tyrosine kinase receptors including VEGFR (vascular endothelial growth factor receptor), PDGFR (platelet‐derived growth factor receptor), and c‐Kit (Llovet *et al*., 2008). Sustained sorafenib exposure induces the production of HGF, and upregulation of c‐Met and p‐Met, which in turn activates its downstream factors, Akt and ERK (Peters and Adjei, 2012). Exposure of HCC cells to sorafenib results in an increase in miR‐21 expression, a decrease in PTEN expression, and sequential Akt activation, leading to upregulation and activation of mammalian target of rapamycin (mTOR) and glycogen synthase kinase‐3β (GSK3β) (He *et al*., 2015; Zhai *et al*., 2014). GSK3β regulates apoptotic proteins (Serova *et al*., 2013), while mTOR controls autophagy by regulating autophagic proteins LC3 and Beclin‐1 (Sini *et al*., 2010). Autophagy switches from a cytoprotective role to a death‐promoting mechanism, and therefore, promoting autophagy leads to increased apoptosis in sorafenib‐resistant HCC cells (Zhai *et al*., 2014). Inhibition of Akt by MK2206 or GDC0068 induces autophagic cell death (Zhai *et al*., 2014), but also upregulates p‐Met via a negative feedback mechanism (Chandarlapaty *et al*., 2011). Capmatinib or cabozantinib inhibits c‐Met, thus leading to the inhibition of the ERK and Akt pathways (Peters and Adjei, 2012) and sequential downregulation of downstream factors controlling cell apoptosis, autophagy, and cell cycle arrest. The c‐Met inhibitors also block the compensatory feedback activation induced by Akt inhibition and cooperate with Akt inhibitors to suppress sorafenib‐resistant HCC cells. In the present study, MK2206 exhibited a stronger proapoptotic effect than capmatinib, while capmatinib displayed a more powerful inhibitory activity on cell proliferation than MK2206. This could be explained by the fact that Akt directly regulates apoptotic proteins, while c‐Met indirectly regulates apoptosis via the Akt pathway. On the other hand, c‐Met regulates proteins involved in cell proliferation via the ERK pathway (Fig. 7).

MK2206 and capmatinib were selected as respective Akt and c‐Met inhibitors based on the promising *in vitro* results, and their favorable activities, potency, selectivity, and tolerance. MK2206 is a highly selective inhibitor of pan‐Akt and is being evaluated in clinical trials for treating solid tumors including HCC and shown reasonably well tolerated (Gupta *et al*., 2015). Capmatinib (formerly known as INC280) is an oral c‐Met inhibitor by selectively disrupting c‐Met *via* competing reversibly for the ATP‐binding site with more than 10 000‐fold selectivity over other kinases (Krepler *et al*., 2016). Capmatinib is also being evaluated in clinical trials for several types of advanced solid tumors including HCC (http://clinicaltrials.gov).

Despite recent progress in the anticancer campaign, the development of molecular targeted drugs for HCC has lagged behind the greater efficacy achieved in some other forms of cancer. Up to now, no distinctive ‘driver gene’ for HCC cells has been identified, and as a result, no drug targeting a single molecule has resulted in significant benefits for patients with HCC (Bruix and Sherman, 2011). Therefore, present strategies to combat HCC have to target the network of a few molecules or pathways. This may explain that sorafenib, a multitargeted tyrosine kinase inhibitor, could stand out as the first effective drug for the treatment of HCC (Cheng *et al*., 2009; Llovet *et al*., 2008). Given that no second‐line drugs are available after the failure of sorafenib (Chan *et al*., 2016), the results presented herein warrant clinical investigation of dual inhibition of c‐Met and Akt pathways, such as the combination of MK2206 and capmatinib, particularly as a second‐line therapy for advanced HCC that becomes acquired resistant to sorafenib.

## Author contributions

XS and HL designed the project, supervised the study and finalized the manuscript; PH performed experiments, analyzed the data and drafted the manuscript. XJ, BZ, GT and DZ participated in experiments, acquired and analyzed the data; HQ, BL and HJ interpreted the data, and contributed to study design and manuscript revision; PH and HL contributed equally to this work.

## Supporting information


**Appendix S1.** Supplementary materials and methods.
**Fig. S1.** Sorafenib‐resistant HCC cells are refractory to sorafenib‐induced growth inhibition and apoptosis.
**Fig. S2.** Inhibition of c‐Met by capmatinib and Akt inhibition by MK2206 are less effective in suppressing parental HCC cells.
**Fig. S3.** Inhibition of c‐Met by cabozantinib enhances the sensitivity of sorafenib‐resistant HCC cells to sorafenib.
**Fig. S4.** Autophagy assay by monodansycadaverine (MDC) staining. Huh7‐SR and HepG2‐SR cells were incubated for 48 h with capmatinib (2 nm), or MK2206 (1 μm) or the combination.
**Fig. S5.** Dual inhibition of Akt and c‐Met inhibits the proliferation of sorafenib‐resistant HCC cells. Huh7, Huh7‐SR, HepG2 and HepG2‐SR cells were incubated for 48 h with capmatinib (2 nm), or MK2206 (1 μm) or the combination.
**Fig. S6.** Sorafenib‐resistant tumors responded poorly to sorafenib treatment. (A) Huh7 or Huh7‐SR cells (5 × 10^6^) were subcutaneously inoculated into mice.
**Fig. S7.** Cell proliferation, apoptosis and gene expression *in vivo*.
