# Supplementary material for: Dual inhibition of Akt and c‐Met as a second‐line therapy following acquired resistance to sorafenib in hepatocellular carcinoma cells
Source: Mol Oncol. 2017 Feb 17;11(3):320–34. doi: 10.1002/1878-0261.12039 (PMC5527443; doi:10.1002/1878-0261.12039)
Supplement: Supplementary file 1 — Appendix S1. Supplementary materials and methods. Fig. S1. Sorafenib‐resistant HCC cells are refractory to sorafenib‐induced growth inhibition and apoptosis. Fig. S2. Inhibition of c‐Met by capmatinib and Akt inhibition by MK2206 are less effective in suppressing parental HCC cells. Fig. S3. Inhibition of c‐Met by cabozantinib enhances the sensitivity of sorafenib‐resistant HCC cells to sorafenib. Fig. S4. Autophagy assay by monodansycadaverine (MDC) staining. Huh7‐SR and HepG2‐SR cells were incubated for 48 h with capmatinib (2 nm), or MK2206 (1 μm) or the combination. Fig. S5. Dual inhibition of Akt and c‐Met inhibits the proliferation of sorafenib‐resistant HCC cells. Huh7, Huh7‐SR, HepG2 and HepG2‐SR cells were incubated for 48 h with capmatinib (2 nm), or MK2206 (1 μm) or the combination. Fig. S6. Sorafenib‐resistant tumors responded poorly to sorafenib treatment. (A) Huh7 or Huh7‐SR cells (5 × 10^6^) were subcutaneously inoculated into mice. Fig. S7. Cell proliferation, apoptosis and gene expression in vivo. [file MOL2-11-320-s001.docx]

**Supplementary Materials for:**

**Dual inhibition of Akt and c-Met as a second-line therapy suppresses sorafenib-resistant hepatocellular carcinoma cells**

Peng Han, Hali Li, Xian Jiang, Bo Zhai, Gang Tan, Dali Zhao, Haiquan Qiao, Bing Liu, Hongchi Jiang, Xueying Sun

**Contents**

**Supplementary Materials and Methods** ........................................................... 3

Antibodies and reagents ................................................................................... 3

Cell proliferation analysis..................................................................................3

Assessment of cell cycle and apoptosis *in vitro*.................................................4

Autophagy assays..............................................................................................4

Transfection of Akt siRNA................................................................................5

Enzyme-linked immunosorbent assay (ELISA)................................................5

Immunoblotting analysis....................................................................................6

Immunohistochemistry and *In situ* Ki-67 proliferation index............................7

*In situ* detection of apoptotic cells......................................................................7

**Supplementary Figures.**.......................................................................................8

Figure S1............................................................................................................8

Figure S2............................................................................................................9

Figure S3............................................................................................................10

Figure S4............................................................................................................11

Figure S5............................................................................................................12

Figure S6............................................................................................................13

Figure S7............................................................................................................14

**Supplementary Materials and Methods**

**Antibodies and reagents**

The antibodies (Abs) against c-Met (ab51067), p-c-Met (Thr1349) (ab47606) and Ki67 (ab15580) were from Abcam (Cambridge, MA, USA). Abs against Akt (C67E7), p-Akt (Ser473) (D9E), ERK (extracellular signaling-regulated kinase) (137F5), phosphorylated ERK (p-ERK) (Thr202/Thyr204) (D13.14.4E), S6K (ribosomal protein S6 kinase), phosphorylated S6K (p-S6K) (Thr389), 4EBP1 (eukaryotic translation initiation factor 4E-binding protein 1 (4EBP1), phosphorylated 4EBP1 (p-4EBP1) (Ser65), Caspase-3 (3G2), LC3 (microtubule-associated protein 1 light chain 3) (D50G8) and Beclin 1 (D40C5) were purchased from Cell Signaling Technology (Danvers, USA). The Abs against PTEN (A2B1), HGF (H-10), cyclin D1 (72-13G) and β-actin (N-21) were from Santa Cruz Biotechnology (CA, USA). Sorafenib, cabozantinib, capmatinib, GDC0068 and MK2206 and were from Jinan Trio Pharmatech Co., Ltd, and were dissolved in dimethyl sulfoxide to make a stock solution of 10 mM. For animal experiments, MK2206 and capmatinib were suspended in the vehicle solution containing Cremophor (Sigma-Aldrich), 95% ethanol and water in a ratio of 1:1:6, The PI (propidium iodide)/Annexin V-FITC apoptosis detection kit was from BD Biosciences. The Cell Counting Kit-8 (CCK-8) kit was from Dojindo Molecular Technologies, Gaithersburg, MD, USA). Terminal deoxynucleotidyl transferase-mediated dUTP nick end labeling agent (TUNEL) was from Roche.

**Cell proliferation analysis**

Cells were seeded into a 96-well plate (3×10^3^/well) in triplicate and cultured overnight. The culture medium was replaced with fresh FBS-free media containing vehicle or testing reagents at various concentrations. Cell viability was measured with a CCK-8 kit. Untreated cells served as controls. Cell viability (%) was calculated according to the formula: experimental OD value/control OD value ×100%.

**Assessment of cell cycle and apoptosis *in vitro***

Cells were seeded at 5.0×10^5^ cells/well in six-well plates and incubated with different reagents for 48 h, and then harvested and counted. A Cell Cycle kit (BD Biosciences, Beijing, China) was used to determine the percentages of cells at different phases of cell cycle by using flow cytometry with a Beckman Coulter Epics Altra II cytometer (Beckman Coulter, California, USA). Cells (1×10^5^) were incubated in 110μl of binding buffer containing 5μl of Annexin V and 5μl of PI for 15 min at room temperature in dark, and then subjected to flow cytometry to measure the apoptosis rate (%) with the cytometer. Or cells were visualized under laser scanning confocal microscopy.

**Autophagy assays**

Cells were incubated with acridine orange (5μM) (Sigma-Aldrich) at 37°C for 15 min, washed with cold PBS, and examined by fluorescent microscopy. AVOs appeared as orange/red fluorescent cytoplasmic vesicles, while nuclei were stained green. Acridine orange-stained cells were further trypsinized and analyzed on a FACScalibur flow cytometer (BD. Biosciences, San Jose, California, USA). The degree of autophagic lysosome was expressed as fold change of acridine orange fluorescence intensity (FL3) of red in treated cells versus control cells. Autophagic vacuoles were also detected with monodansylcadaverine (MDC) (Sigma-Aldrich) staining. Briefly, cells were washed with PBS and then incubated with 0.05 mM MDC in PBS at 37°C for 45 min. After incubation, the cells were washed four times with PBS and immediately analyzed by flow cytometry, and viewed by fluorescence microscopy.

**Transfection of Akt siRNA**

The double-strand Akt-siRNA (5’- GUGGUCAUGUACGAGAUGATT-3' and 5'-UCAUCUCGUACAUGACCACTT-3') targeting human Akt1 (GenBank:　NM_001014431.1), Akt2 (GenBank:　XM_005336494.1) and Akt3 (GenBank: XM_004691046.1) with two introduced thymidine residues at the 3’ end were purchased from GenePharma Co., Ltd. (Shanghai, China). A nonspecific scrambled siRNA 5’-UUCUCCGAACGUGUCACGU-3’ and 5’-ACGUGACACGUUCGGAGA

A-3’) served as a control. Cells were grown to 60-70% confluence, and incubated with siRNAs at a final concentration of 0.1 μM by using Lipofectamine^TM^ 2000 (Invitrogen, Beijing, China) in a serum-free medium for 48 h and then subjected to the assays.

**Enzyme-linked immunosorbent assay (ELISA)**

Cells were seeded into six-well plates in DMEM without FBS. Forty-eight hours later, cultured media were collected. The concentrations of HGF in the media were detected by an HGF Human ELISA Kit (KAC2211; Invitrogen, Carlsbad, CA, USA) following the manufacturer’s instructions.

**Immunoblotting analysis**

Cells or tumor tissues were homogenized in protein lysate buffer (50 mM Tris pH 7.4, 100 μM EDTA, 0.25 M sucrose, 1%SDS, 1% NP40, 1μg/ml leupeptin, 1μg/ml pepstatin A and 100 μM phenyl methyl sulfonyl flouride) and debris was removed by centrifugation at 10,000 × g for 10 min at 4^o^C. Protein concentrations were determined (Bio-Rad, Richmond, CA, USA). Lysates were resolved on sodium dodecyl sulfate-polyacrylamide (SDS-PAGE) gels, electrophoretically transferred to polyvinylidene difluoride (PVDF) membranes. The membranes were blocked in TBST (137 mM NaCl, 20 mM Tris HCl [pH 7.6], and 0.1% [v/v] Tween 20) containing 5% (w/v) nonfat dry milk at 37^o^C for 2 h, and then incubated overnight with primary Abs, and subsequently with alkaline phosphatase-conjugated secondary Abs for 2 h at room temperature in the dark. They were developed with 5-bromo-4-chloro-3-indolyl phosphate (BCIP)/ nitro blue tetrazolium (NBT) (Tiangen Biotech Co. Ltd., Beijing, China). The density of each band was measured using a densitometric analysis program (FR200, Shanghai, China). In preliminary experiments, serial dilutions of lysates (containing 2.5, 5, 10, 20, 40 or 80µg protein) were immunoblotted; band intensities were measured and plotted against protein amounts to generate a standard curve, and the amount of protein for each blot was determined.

**Immunohistochemistry and In situ Ki-67 proliferation index**

Formalin fixed tumor specimens were transferred to 70% ethanol and subsequently paraffin-embedded and sectioned. Tumor sections were rinsed with PBS, blocked with 3% BSA for 2 h, and incubated with Abs against Ki-67, p-Akt, p-Met, or cleaved caspase-3 at 4^o^C overnight. They were subsequently incubated for 30 min with the appropriate secondary Ab using the Ultra-Sensitive TMS-P kit (Zhongshan Co., Beijing, China), and immunoreactivity developed with Sigma FAST DAB (3,3’-diaminobenzidine tetrahydrochloride) and CoCl_2_ enhancer tablets (Sigma-Aldrich, Shanghai, China). Sections were counterstained with hematoxylin, mounted, and examined by microscopy. The Ki-67 positive cells were counted in 10 randomly selected × 400 high-power fields under microscopy. The Ki-67 proliferation index was calculated according to the following formula: the number of Ki-67 positive cells/ the total cell count × 100%.

**In situ detection of apoptotic cells**

The above tumor sections were stained with the TUNEL (Terminal deoxynucleotidyl transferase dUTP nick end labeling) (Roche, Shanghai, China). The TUNEL positive cells were counted in 20 randomly selected × 200 high-power fields under microscopy. The apoptosis index was calculated according to the following formula: the number of apoptotic cells × /total number of nucleated cells × 100%.

**Supplementary Figures**


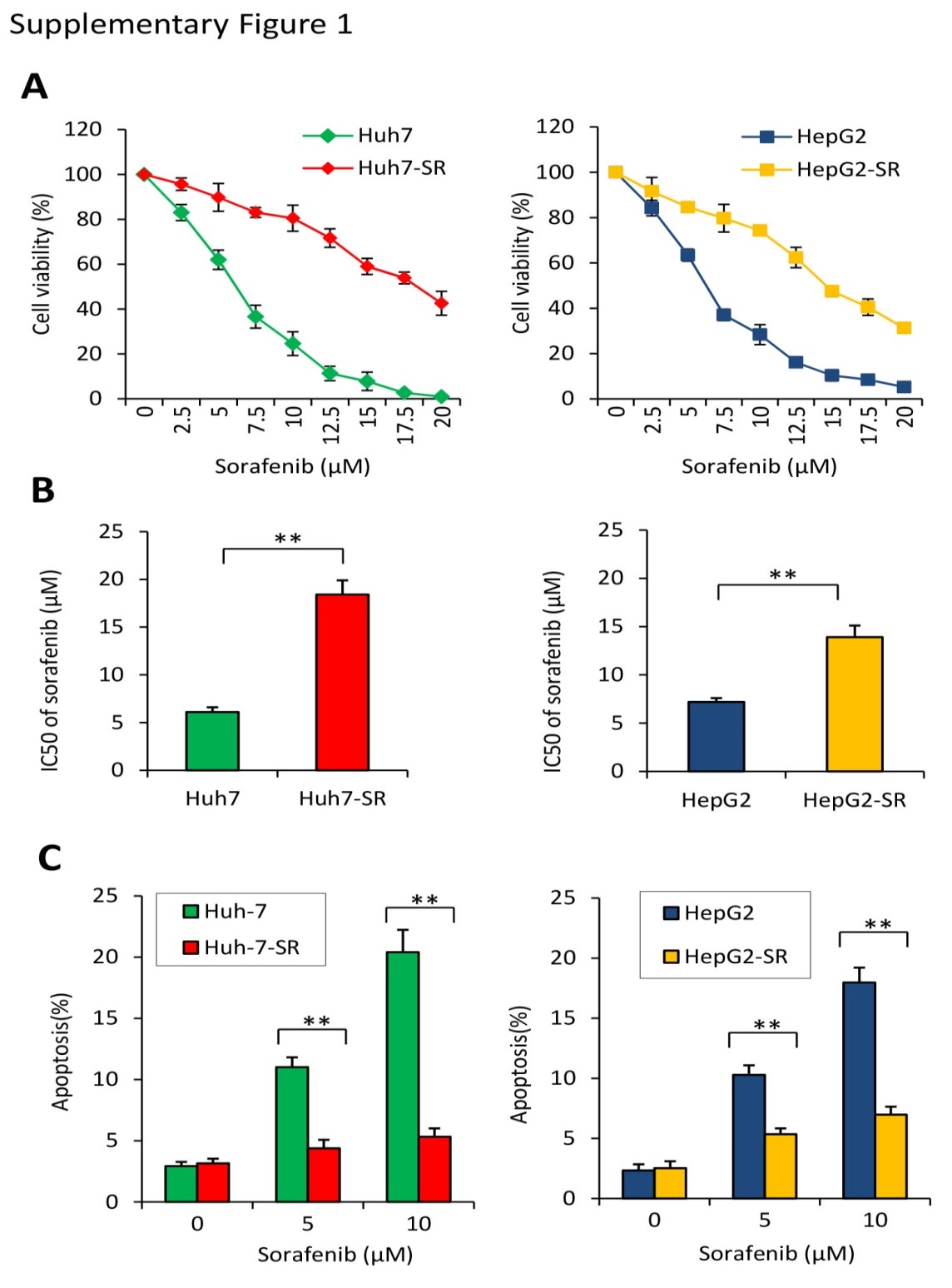


Supplementary Figure S1 - Sorafenib-resistant HCC cells are refractory to sorafenib-induced growth inhibition and apoptosis. (A) Huh7, Huh7-SR, HepG2 and HepG2-SR cells were incubated for 48 h with various concentrations of sorafenib. Cell viability (%) was normalized to respective untreated cells. (B) The values of IC_50_ for each cell line were calculated. (C) The above cells incubated with 0, 5 or 10μM of sorafenib were analyzed cytometrically to determine cell apoptosis (%). “**” (P<0.001) indicates a significant difference.


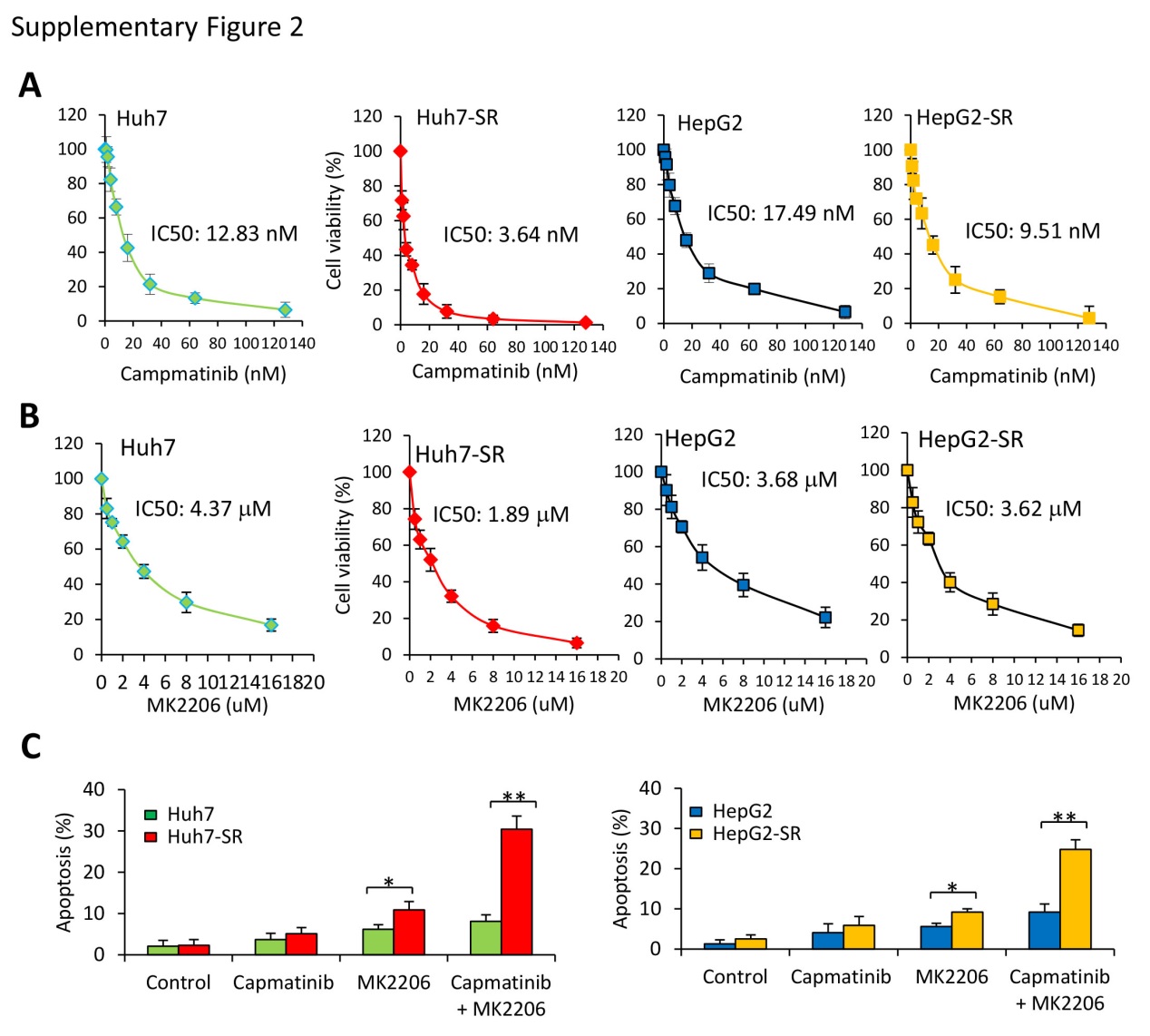


Supplementary Figure S2 - Inhibition of c-Met by capmatinib and Akt inhibition by MK2206 are less effective in suppressing parental HCC cells. (A, B) Huh7, Huh7-SR, HepG2 and HepG2-SR cells were cultured for 48 h with various concentrations of capmatinib (A) or MK2206 (B), and subjected to cell viability assays. The values of half maximal inhibitory concentration (IC50) were calculated. (C, D) Cells were incubated for 48 h with capmatinib (2 nM), or MK2206 (1 μM) or their combination, and subjected to apoptosis assay. “*” (P<0.05) and “**” (P<0.001) indicate a significant difference.


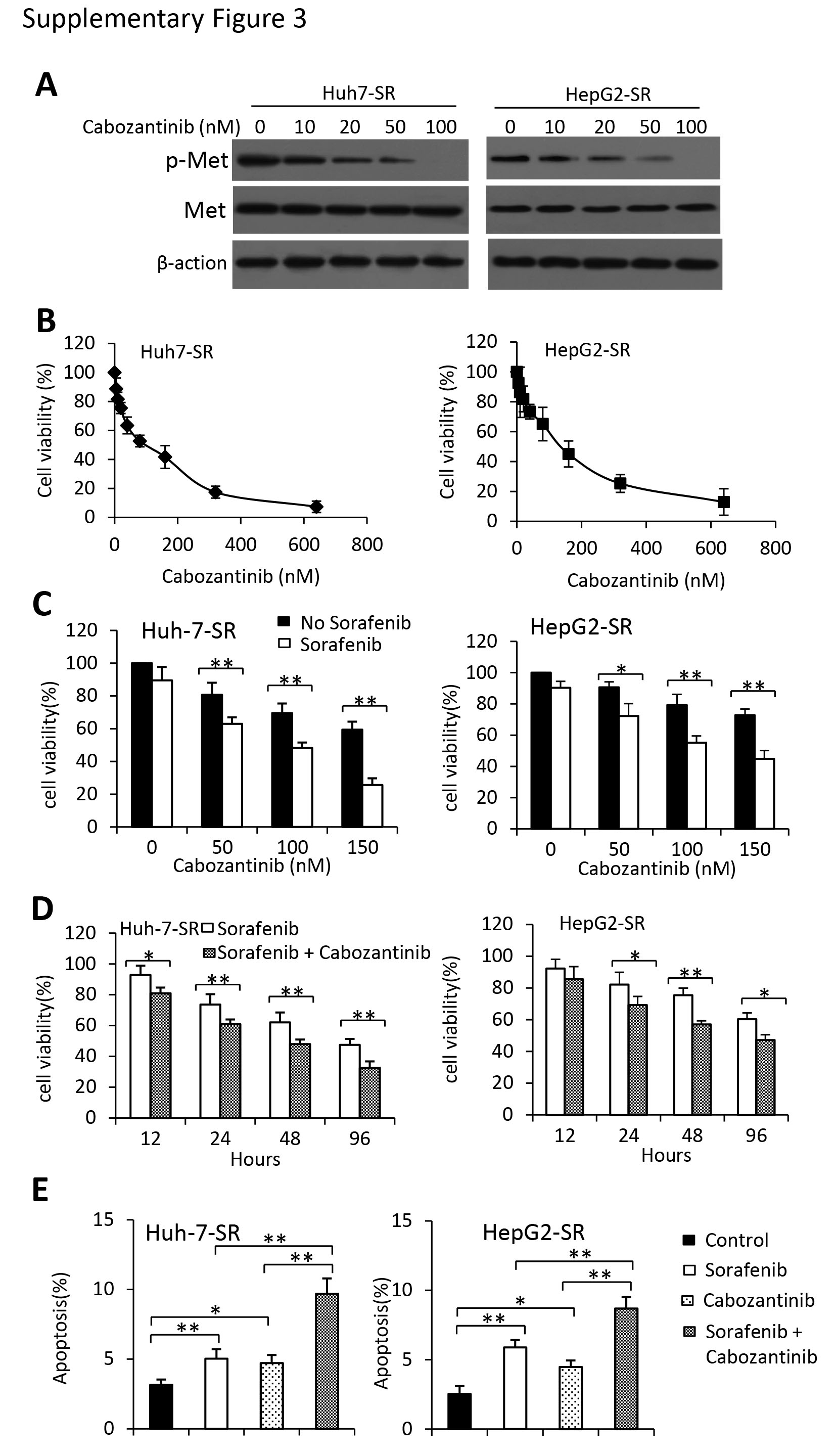


Supplementary Figure S3 - Inhibition of c-Met by cabozantinib enhances the sensitivity of sorafenib-resistant HCC cells to sorafenib. (A, B) Huh7-SR and HepG2-SR cells were incubated for 24 h to various concentrations of cabozantinib, and subjected to immunoblotting (A) or cell viability (B) assays. (C) Cells were incubated for 48 h with various concentrations of cabozantinib in the presence or absence of sorafenib (5 μM). (D) Cells were incubated with cabozantinib (100 nM) in the presence or absence of sorafenib (5 μM) and harvested at indicated time points. (B-D) Cell viability (%) was normalized to the respective untreated cells. (E) Cells were incubated for 48 h with sorafenib (5μM), cabozantinib (100 nM) or the combination. Cell apoptosis (%) was analyzed by flow cytometry. “*” (P<0.05) and “**” (P<0.001) indicate a significant difference.


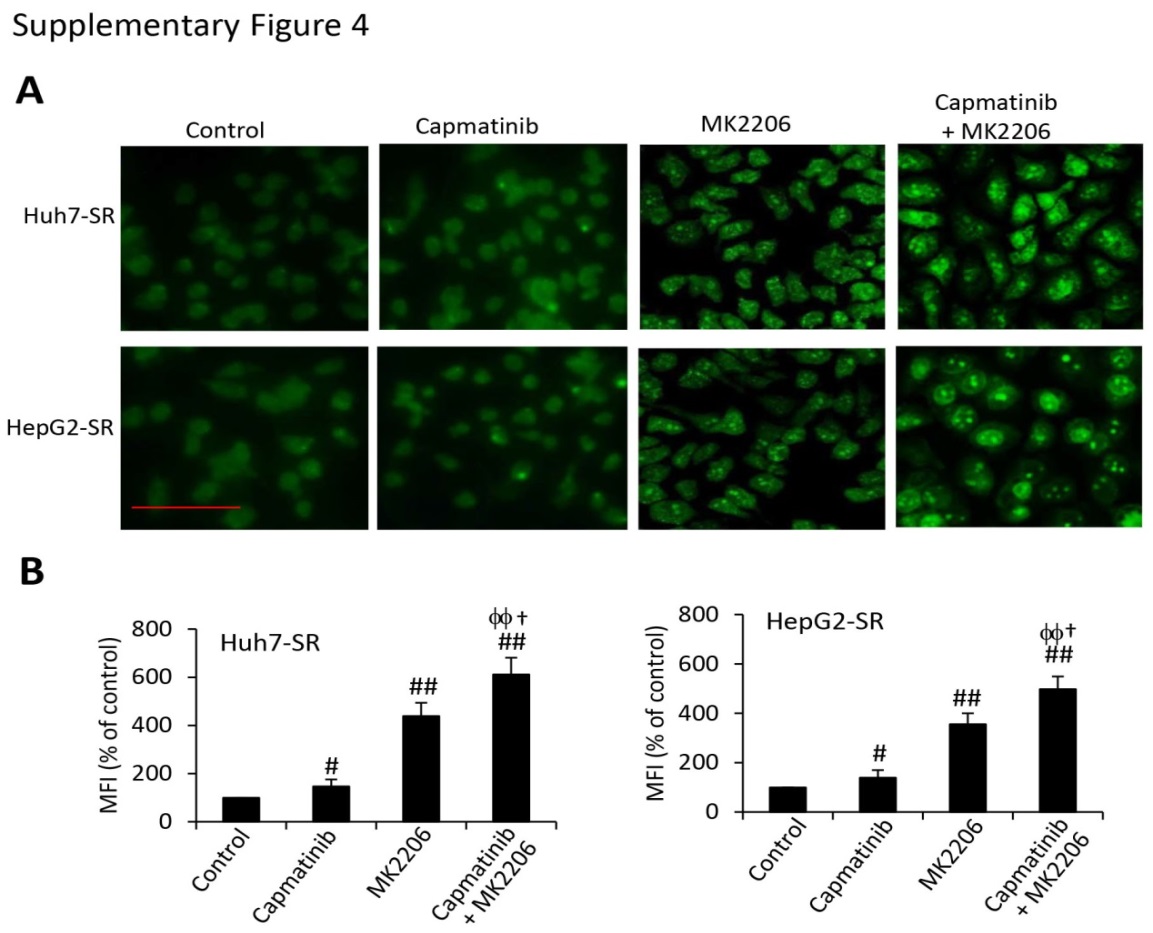


Supplementary Figure S4 - Autophagy assay by monodansycadaverine (MDC) staining. Huh7-SR and HepG2-SR cells were incubated for 48 h with capmatinib (2 nM), or MK2206 (1 μM) or the combination. (A) Representative images were taken from the cells stained by MDC (Original magnification × 400, scale bar =500 μm). (B) The mean fluorescence intensity (MFI) (% of control) was measured by flow cytometry. Untreated cells served as controls. “#” (P<0.05) and “##” (P<0.001) indicate a significant increase vs. controls. “φφ” (P<0.001) vs. capmatinib alone; “†” (P<0.05) vs. MK2206 alone.


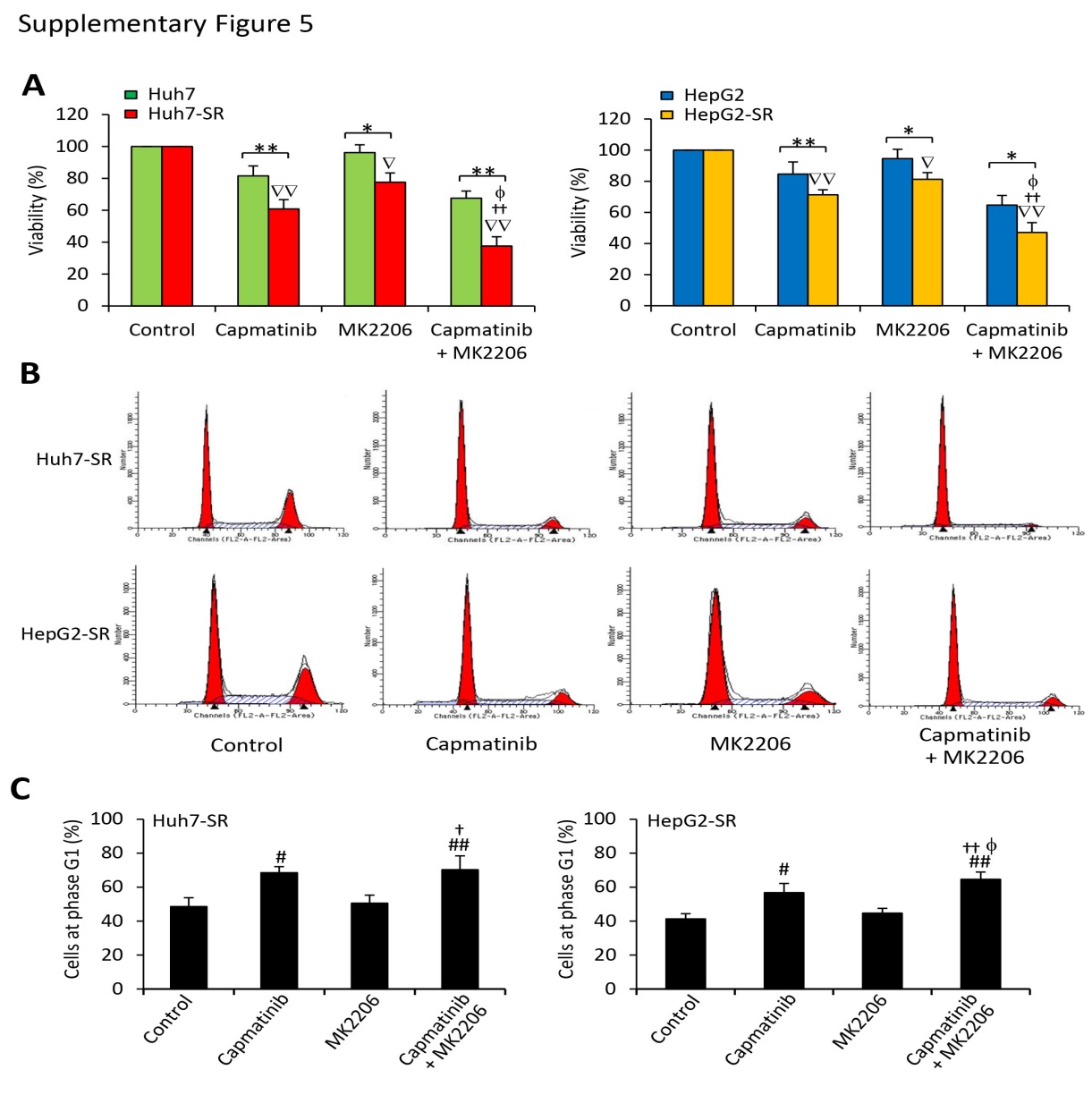


Supplementary Figure S5 - Dual inhibition of Akt and c-Met inhibits the proliferation of sorafenib-resistant HCC cells. Huh7, Huh7-SR, HepG2 and HepG2-SR cells were incubated for 48 h with capmatinib (2 nM), or MK2206 (1 μM) or the combination. (A) Cell viability (%) was normalized to untreated controls. (B) Cell cycle distribution was measured by flow cytometry. (C) The percentages of cells at the G1 phase were plotted. “*” (P<0.05) and “**” (P<0.001) indicate a significant difference. “#” (P<0.05) and “##” (P<0.001) indicate a significant increase, while “∇” (P<0.05) and “∇∇” (P<0.001), a significant reduction, versus controls. “φ” (P<0.05) vs. capmatinib alone; “†” (P<0.05) and “††” (P<0.001) vs. MK2206 alone.


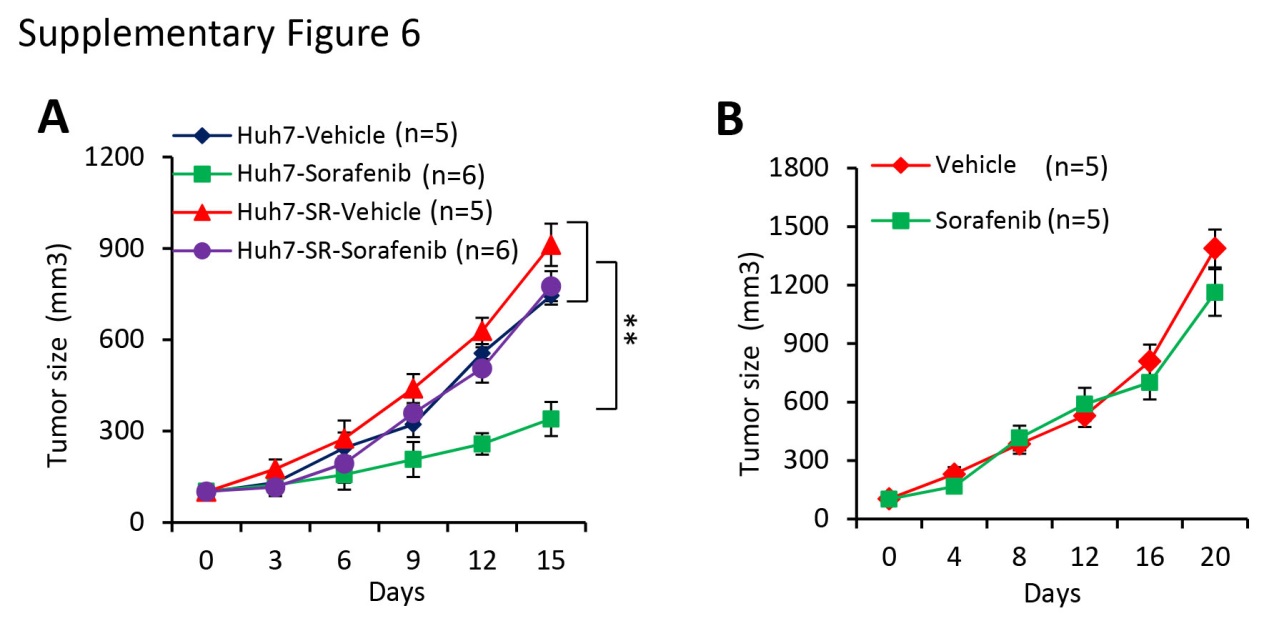


Supplementary Figure S6 - Sorafenib-resistant tumors responded poorly to sorafenib treatment. (A) Huh7 or Huh7-SR cells (5×10^6^) were subcutaneously inoculated into mice. Two weeks later, when tumors grew to ~100 mm^3^, the mice were assigned to different groups and received daily oral administration of vehicle or 30 mg/kg sorafenib for 15 days as described previously (Zhai et al., 2014). Tumor volumes were measured every 3 days. (B) Huh7-SR tumors for second-line therapies were established as described in Figure 6A. The mice were assigned to two groups and received daily oral administration of vehicle or sorafenib at a dose of 30 mg/kg, respectively. The tumor volumes were measured every 4 days. “**” (*P*<0.001) indicate a highly significant difference.


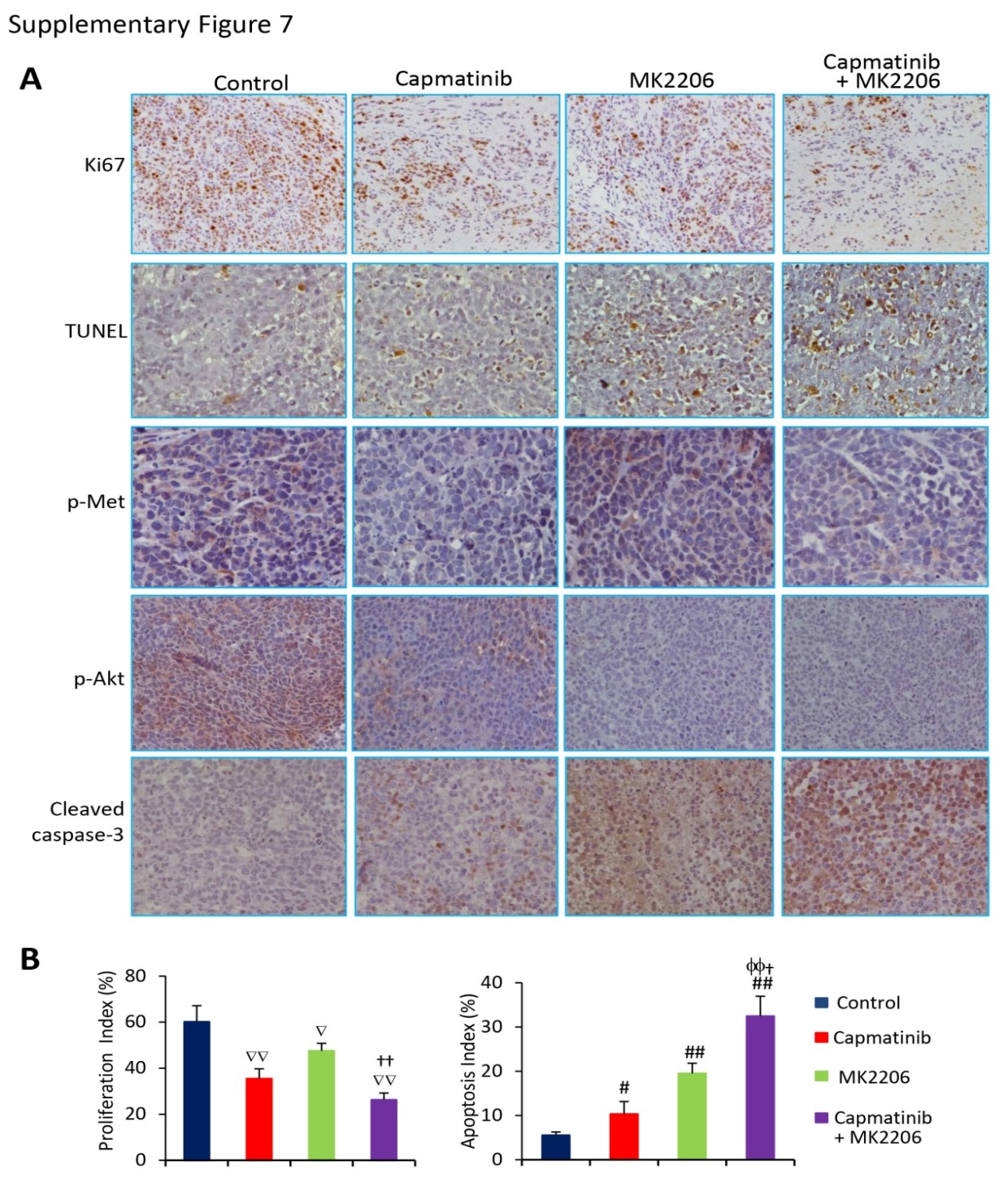


Supplementary Figure S7 - Cell proliferation, apoptosis and gene expression *in vivo*. (A) Representative images of tumor sections taken from Figure 6 were immunostained with an anti-Ki67 Ab (magnification × 200), TUNEL (magnification × 400), anti-p-c-Met Ab (magnification × 400), anti-p-Akt Ab (magnification × 200) or anti-cleaved caspase-3 Ab (magnification × 200). (B) Proliferation index (%) and apoptosis index (%) and were quantified. “#” (P<0.05) and “##” (P<0.001) indicate a significant increase; while “∇” (P<0.05) and “∇∇” (P<0.001), a significant reduction, versus controls. “φφ” (P<0.001) vs. capmatinib alone; “†” (P<0.05) and “††” (P<0.001) vs. MK2206 alone.
